# Supplementary material for: Randomized Clinical Trials and Observational Tribulations: Providing Clinical Evidence for Personalized Surgical Pain Management Care Models
Source: J Pers Med. 2023 Jun 25;13(7):1044. doi: 10.3390/jpm13071044 (PMC10381640; doi:10.3390/jpm13071044)
Supplement: Supplementary file 1 [file jpm-13-01044-s001.zip › jpm-2403747-SI.pdf]

Supplementary Document S1.

Interamerican Society For Minimally Invasive Spine Surgery—La Sociedad Interamericana de Cirugía de Columna Mínimamente Invasiva (SICCMI)

Kai-Uwe Lewandrowski
Morgan P. Lorio
Álvaro Dowling
Paulo Sérgio Teixeira De Carvalho

The International Intradiscal Therapy Society (IITS.org)

Kai-Uwe Lewandrowski
Anthony Yeung

Brazilian Society For Thoracic Surgery—Sociedade Brasileira de Cirurgia Torácica (SBCT)

Rossano Kepler Alvim Fiorelli

International Society for Minimal Intervention in Spinal Surgery (ISMISS)

Benedikt W. Burkhardt
Joachim M. Oertel

the Chinese Orthopaedic Association—Minimally Invasive Surgery Section (COA-MIS Section)

Huilin Yang

the Iberolatinoamerican Spine Society—La Sociedad Iberolatinoamericana De Columna (SILACO)

Jorge Felipe Ramírez León
Jaime Moyano

Brazilian Society of Neurosurgery—Sociedade Brasileira de Neurocirurgia (SBNC)

Roth AA Vargas
